# Saccade adaptation deficits in developmental dyslexia suggest disruption of cerebellar-dependent learning

**DOI:** 10.1186/s11689-017-9218-5

**Published:** 2017-11-09

**Authors:** Edward G. Freedman, Sophie Molholm, Michael J. Gray, Daniel Belyusar, John J. Foxe

**Affiliations:** 1 The Del Monte Institute for Neuroscience, Department of Neuroscience, University of Rochester School of Medicine and Dentistry, Rochester, NY 14642 USA; 2 The Sheryl and Daniel R. Tishman Cognitive Neurophysiology Laboratory, Department of Pediatrics, Albert Einstein College of Medicine & Montefiore Medical Center, Bronx, NY 10461 USA; 3 The Dominic P. Purpura Department of Neuroscience, Albert Einstein College of Medicine, Bronx, NY 10461 USA; 4 The Graduate Center of the City University of New York, New York, NY 10031 USA

**Keywords:** Dyslexia, Eye movements, Adaptation, Saccades, Cerebellum, Reading

## Abstract

**Background:**

Estimates of the prevalence of developmental dyslexia in the general population range from 5% to as many as 10%. Symptoms include reading, writing, and language deficits, but the severity and mix of symptoms can vary widely across individuals. In at least some people with dyslexia, the structure and function of the cerebellum may be disordered. Saccadic adaptation requires proper function of the cerebellum and brainstem circuitry and might provide a simple, noninvasive assay for early identification and sub-phenotyping in populations of children who may have dyslexia.

**Methods:**

Children between the ages of 7 and 15 served as participants in this experiment. Fifteen had been diagnosed with developmental dyslexia and an additional 15 were typically developing children. Five of the participants diagnosed with dyslexia were also diagnosed with an attention deficit hyperactivity disroder and were excluded from further analyses. Participants performed in a saccadic adaptation task in which visual errors were introduced at the end of saccadic eye movements. The amplitudes of primary saccades were measured and plotted as a function of the order in which they occurred. Lines of best fit were calculated. Significant changes in the amplitude of primary saccades were identified.

**Results:**

12/15 typically developing children had significant adaptation of saccade amplitude in this experiment. 1/10 participants with dyslexia appropriately altered saccade amplitudes to reduce the visual error introduced in the saccade adaptation paradigm.

**Conclusions:**

Proper cerebellar function is required for saccadic adaptation, but in at least some children with dyslexia, cerebellar structure and function may be disordered. Consistent with this hypothesis, the data presented in this report clearly illustrate a difference in the ability of children with dyslexia to adapt saccade amplitudes in response to imposed visual errors. Saccadic adaptation might provide a noninvasive assay for early identification of dyslexia. Future work will determine whether reduced saccadic adaptation is pervasive in dyslexia or whether this identifies a sub-phenotype within the larger population of people identified with reading and language deficits.

## Background

### Eye movements in dyslexia

Language, writing, and reading deficits are principle elements in identifying children with developmental dyslexia. However, a much broader range of behavioral differences can be observed. Motor learning deficits, discoordination, imbalance, and reduced multisensory and motor processing speeds are often observed [1–7], and several studies of eye movements have revealed differences in saccadic control in dyslexia [8–13]. For example, a large population study demonstrated reduced performance in an anti-saccade task [8], and in a study that employed a double-step eye movement task, increased latencies of orthogonal (but not co-linear) second saccades were observed in dyslexic participants [13]. While these studies provide clues about neural mechanisms that may be altered in dyslexia, neither anti-saccade deficits nor problems in the context of a double-step task are proximal causes of reading disturbances in children with dyslexia. On the other hand, fine control of saccade accuracy and precision is clearly of critical importance to fluent reading [14]. In children with dyslexia, if saccades across text do not land at the expected locations, text might well appear to shift relative to the reader rather than remaining stationary as it will when eye movements land reliably at the commanded positions. Similarly, imprecise eye movements might require multiple corrective saccades to progress along a line of text or to move easily from the end of one line to the beginning of the next. Disruption of these movements will prolong reading times and make comprehension more difficult. It is instructive that reading without needing to make saccades can increase reading rates by 30–40% without degrading comprehension [15], suggesting that even in typically developing children saccades may limit reading rates.

### Saccadic adaptation and the cerebellum

When saccades become systematically inaccurate due to eye muscle weakness or, in the lab, by artificially shifting the visual input using prism goggles, the cerebellum and brainstem circuitry operate to reduce the resultant errors [16–19]. Visual errors can also be experimentally introduced during or just after a saccadic eye movement [16, 20–31]. When such errors are persistent, the cerebellar circuitry operates to alter the amplitude of saccades such that the resultant errors are reduced [20, 32–34]. Thus, visual errors trigger cerebellar and brainstem control circuits to rapidly adjust the movement commands in order to maintain saccade accuracy. Short-term adaptation tasks in which a visual error is surreptitiously introduced provide a systematic way to evaluate the function of the circuits involved in the neural control of visual orienting. A failure of the cerebellum in this regard can be revealed in the inability to alter saccade amplitudes leaving saccades inaccurate [18, 35–41].

### Cerebellum and dyslexia

The data on structural differences in the cerebella of people diagnosed with dyslexia are somewhat variable ([42–46]). For instance, Brambati [43] found consistent reductions in the volume of gray matter in the deep cerebellar nuclei, whereas Brown et al. [47] reported reduced gray matter in the right hemisphere of lobe VII of dyslexics compared to the left hemisphere (in right-handed participants). Rae et al. [48] also reported a distinct left-right difference in cerebellar hemispheres in dyslexics. They found that in control participants (“good readers”), there was a clear right > left asymmetry in the cerebellar hemispheres but that this laterality difference was not seen in their dyslexic participants. Post-mortem studies [49] report fewer small cells and more large cells in the medio-posterior cerebellum (lobules VI and VII), and Eckert and colleagues [50] could correctly characterize 72% of dyslexics and 88% of controls based on a cerebellar structural model.

Neural activity in lobes VI and VII of the cerebellum has also been studied in people with dyslexia. Nicolson et al. [53] showed reduced right lobe VI activity during an implicit motor learning task in adults with dyslexia. This group has also reported anatomical differences in the olivo-cerebellar pathway in adults with dyslexia [49]. Menghini et al. [51] reported altered lobe VI activity during motor learning, but in this case, the activity was higher in dyslexia compared to controls. This region of the cerebellum is critical for maintenance of saccade accuracy (endpoint error) and precision (endpoint variability). Further evidence of disordered cerebellar activity in dyslexia is provided by the eyeblink conditioning experiment of Nicolson and colleagues [52]. Based on these and other data, Nicolson and Fawcett [7, 53] proposed that cerebellar dysfunction that exists from birth may be a root cause of the symptoms of dyslexia. One prediction of this cerebellar-dyslexia hypothesis is that behaviors which depend crucially on the proper functioning of the cerebellum will be reduced or missing in people with dyslexia. To test this, we investigated saccadic adaptation in people with dyslexia to determine if, in response to persistent visual error, they are able to alter the amplitude of saccades. As outlined above, saccadic adaptation is well known to depend on an intact cerebellum. In this study, as in many previous adaptation studies, a visual error is introduced by moving the target after a saccade has been initiated. This saccadic adaptation task [20] reliably leads to systematic changes in saccade amplitudes in typically developing humans as well as non-human primates [16, 22, 27, 29, 54] and depends on the proper function of the posterior cerebellar vermis [18, 37, 55, 56]. It may function as a new and powerful tool for identifying dyslexia that arises from a disordered cerebellum.

## Methods

### Participants

Fifteen individuals (5 females; 1 left-handed; mean age = 10.9 ± 2.8) diagnosed with dyslexia (DYS) served as participants along with 15 typically developing (TD), age-matched control participants (7 females; mean age = 12.5 ± 2.8). Verbal, performance, and full-scale IQ tests were given to each participant. Although there were no statistically significant differences in either the VIQ or the PIQ measures, mean FSIQ was significantly higher in the TD population compared to the DYS (Student’s *t* test: *t* = 2.45; *p* < 0.05). Five participants with dyslexia were also diagnosed as having ADHD; the data from these participants is excluded from further analyses. Four of the remaining 10 participants with dyslexia were involved in or had participated in remediation of reading and/or math skills. The Woodcock-Johnson (WJ) Letter-Word Identification, the WJ Reading Fluency, and the WJ Word Attack Tests were also given to the participants with dyslexia. These tests are scored such that the mean and standard deviation of the typically developing population is 100 ± 15. The DYS participants in this experiment had the following scores: Letter Word (92.8 ± 19.13), Reading Fluency (83.1 ± 15.45), Word Attack (90.7 ± 8.64). See Table 1 for details. Mean (SD) age of TD participants was 12.5 (2.8) (Table 2). Mean (SD) age of DYS participants was 11.13 (3.34). There was not a significant difference in participant ages (KS test *p* = 0.57).

**Table 1:** Participants with dyslexia

Participant	Age	Gender	VIQ	PIQ	FSIQ	Word ID	WJ-R	WJ-WA	ADHD	Adapt
1	8.4	m	133	112	126	100	83	91	n	n
2	7.7	m	85	99	90	104	99	109	n	n
3	15.7	f	104	102	104	93	67	88	n	y
4	7.4	m	91	108	100	99	95	99	n	n
5	14	f	91	76	88	76	81	86	n	n
6	10.5	m	102	109	106	86	88	94	n	n
7	11.2	m	83	86	82	53	47	83	n	n
8	15.2	f	114	110	114	104	90	91	n	n
9	13.8	f	112	134	125	109	89	88	n	n
10	7.4	m	104	88	96	104	92	78	n	n
Mean	11.13		101.9	102.4	103.1	92.8	83.1	90.7		
SD	3.34		15.29	16.35	15.04	19.13	15.45	8.64		

Wechsler Scale *VIQ* verbal IQ, *PIQ* performance IQ, *FSIQ* full-scale IQ, *Word ID* Woodcock-Johnson Word Identification, *WJ-R* Woodcock-Johnson Reading, *WJ-WA* Woodcock-Johnson Word Attack, *ADHD* attention deficit hyperactivity disorder status, *Adapt* significant saccade adaptation (y/up identifies subject that adapted significantly but in the incorrect direction)

**Table 2:** Typically developing participants

Participant	Age	Gender	VIQ	PIQ	FSIQ	Adapt
1	15.7	m	121	132	130	y
2	14.1	f	99	115	107	y
3	10.2	f	134	110	126	y
4	15	m	108	104	107	y
5	15.2	m	99	106	104	y
6	8.3	f	97	101	93	y
7	13.2	f	94	108	101	y
8	12.6	m	113	106	111	y
9	9.3	m	114	98	107	n
10	11.2	f	134	104	122	y
11	15.9	f	123	94	105	y
12	15.9	m	120	131	129	n
13	8.2	m	104	115	109	y
14	13.5	m	140	119	134	y
15	9.7	f	102	117	110	n
Mean	12.5		113.5	110.7	113.0	
SD	2.8		14.8	11.0	12.1	

### Tasks

Visual targets were presented on a computer monitor placed in front of each participant. Initial control trials consisted of presentation of a central target (the “T0” target) that the participants were required to fixate within ± 2° for between 500 and 1500 ms. At the end of this interval, the T0 target was turned off and a new target (T1) was illuminated 12° to either the left or right of the T0 location. Participants were instructed to look at the new target. Targets in other locations were also used in this pre-adaptation epoch. After 40 control trials, adaptation trials began. Adaptation trials were similar to control trials, but as the participant made a saccade to the T1 location, the target was turned off and relocated so that it was only 9° (T2) from the original fixation location (T0). If the participant made an accurate saccade to the T1 location, there would appear to be a 3° overshoot since the target was now at the T2 location. The repeated, surreptitious introduction of a visual error at the end of a saccade can drive sensorimotor adaptation [20].

### Analysis

Eye movements were measured using an EyeLink1000 (SR Research, Ottawa, Canada). Saccades were identified and movement amplitudes (change in position from start to end of marked movements) stored for off-line analysis. Amplitudes of primary saccades (initial movements from T0 to the T1 target location) were measured and plotted as function of the order in which they occurred during the experimental session (trial number). Lines of best fit (least squares) and 95% confidence intervals were calculated. Each participant was categorized as having adapted or having failed to adapt based on the statistical significance of the slope of the line of best fit; no adaptation occurred when the 95% confidence interval of the slope included zero. Ratios of adapters to non-adapters for the TD and DYS groups were then calculated and a *χ* ^2^ test was used to determine whether these ratios differed. In addition, the mean amplitude of the first 10 adaptation trials was compared to the mean amplitude of the last 10 using a *t* test (single tailed, *p* = 0.05). All analyses and statistical tests were accomplished using Matlab (MathWorks, Natick, MA).

## Results

**Fig. 1:**
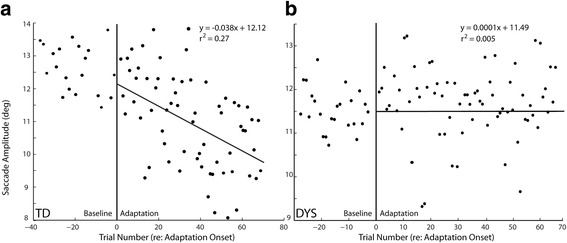
Saccadic adaptation examples. This figure plots the amplitude of primary saccadic eye movements as a function of the trial number in an experimental session. Pre-adaptation, control trials are plotted to the left of the vertical line positioned at 0. To the right are adaptation trials. In **a** (labeled TD), data from one of the typically developing control participants is plotted, along with a line of best fit for the adaptation trials. **b** (labeled DYS) An example session with one of the participants diagnosed with dyslexia. Line of best fit to the adaptation data is also shown

An example of adaptation from one of the TD participants is presented in Fig. 1a. The amplitude of the primary saccade on each trial is plotted in the order that it occurred. Trials to the left of the vertical line are non-adaptation control trials (baseline). Trials to the right of the vertical line are adaptation trials. Before adaptation trials were introduced, this participant made saccades to the T1 target that had a mean amplitude of 12.17 (SD = .65). Within the first 10 or so trials, the mean amplitude of saccades made to this same target begin to decline as a result of the systematic visual error introduced after the saccade ends. This decline in saccade amplitude continues over the next 30 trials, until the mean amplitude of the final 10 saccades was 10.20 (SD = .83). Comparing the mean amplitudes of the first 10 to the last 10 adaptation trials reveals a statistical difference in saccade amplitudes (*t* test: *t* = 5.45, df = 18 *p* < 0.0001). The line of best fit to the adaptation trials is also shown. The slope of this line was statistically different from zero (95% confidence interval (− 0.027, − 0.010) of the slope does not include zero). Compare these data with those plotted in Fig. 1b which illustrate an adaptation session from one of the participants with dyslexia. There is a clear lack of change in saccade amplitude during adaptation trials. The slope of the line of best fit was not statistically different from zero (slope = 0.0001 (− 0.009, 0.017)). In addition, there was no difference in the mean amplitudes of the first and last 10 trials from this participant (*t* test: *t* = 1.5, df = 18, *p* > 0.15). Note in this participant, what appears to be high variability of saccade amplitudes during the adaptation trials. Root mean squared errors (RMSE) of each fit were calculated to determine whether there was a systematic difference in variability of saccade endpoints in the DYS and TD participants. Although the mean RMSE of DYS participants was higher (1.37) compared to TD participants (1.13), it was not a significant difference (KS test *p* = 0.19).

**Fig. 2:**
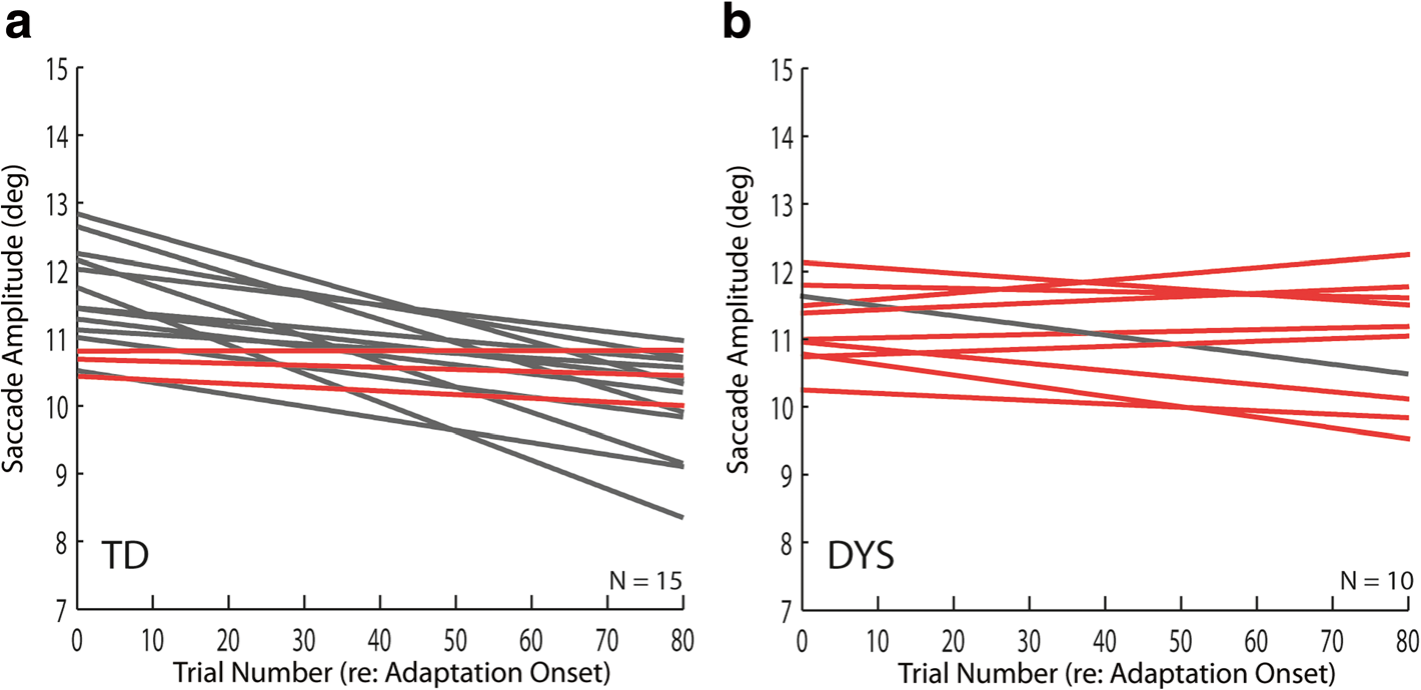
Lines of best fit. All of the lines of best fit for each of the TD (**a**) and DYS (**b**) subjects are superimposed. 95% confidence intervals on the slopes were calculated. Gray lines indicate significant slopes (95% confidence intervals did not include 0). Red lines indicate slopes that were not statistically different from 0. The green line in **b** marks a subject that had a significant increase in saccade amplitude over the course of adaptation trials. Dashed lines indicate participants that also met criteria for a diagnosis of ADHD

Not every typically developing participant adapted as well as the one illustrated in Fig. 1a. Likewise, not every participant with dyslexia failed to adapt. In Fig. 2a, the lines of best fit for each TD participant are superimposed. Those having slopes that were statistically different from zero are plotted in gray (12/15) whereas those with slopes not different from zero are plotted in red (3/15). That some TD participants did not change the amplitude of their saccadic eye movements during an adaptation session is not unusual. In the study by Salman and colleagues [57], 13 of 39 TD participants ranging in age from 8 to 19 years old failed to show significant saccadic adaptation. A similar plot of lines of best fit for each DYS participant is shown in Fig. 2b. In this case, 9/10 participants failed to adapt. When we pooled all the DYS participants, the ratio of adapters to non-adapters in the TD and DYS groups was significantly different (*χ* ^2^ = 11.78, df = 1, *p* < 0.0006). For TD and DYS participants, the amplitudes of the first 10 adaptation trials were compared to the last 10 adaptation trials. In every case, when the slope of the line of best fit was significantly different from zero (gray lines in Fig. 2a, gray and green lines in Fig. 2b), there was a statistical difference in mean saccade amplitudes at the beginning and end of adaptation (*t* test, *p* < 0.05). In every case for which the slope of the best fit line could not be distinguished from zero (red lines in Fig. 2a, b), there was no significant difference in mean saccade amplitudes (*t* test, *p* > 0.05).

## Discussion

Movements, even relatively simple movements like saccades, are not always accurate. If errors persist and are properly detected, the nervous system may attempt to adjust the motor output to reduce or eliminate discrepancies between desired and actual outcomes. Failure to perceive errors or an inability to appropriately modulate motor commands in response to such errors can have drastic behavioral consequences (e.g., disorientation, discoordination, ataxia).

Rapid sensorimotor adaptation of the amplitude of saccadic eye movements has often been investigated by moving the visual target during or just after the end of an ongoing saccadic eye movement [16, 20–22, 24–27, 29, 30, 32, 54, 58–64]. Over the course of repeated trials, the amplitude of the initial saccade can be increased or decreased reducing the residual visual error [20, 32, 34, 60]. The cerebellum is an important mediator of this sensorimotor adaptation [17, 65]. During saccadic adaptation, activity of Purkinje cells in vermis lobules VI and VII and the deep cerebellar nucleus that they project to (caudal fastigial nucleus) are critical [66, 67]. Given the functional and structural differences reported in some people with dyslexia [2, 44, 48, 49, 68, 69], saccadic adaptation has the potential to provide a behavioral window into the functional condition of the cerebellar vermis in populations with developmental disorders.

The data presented in this study clearly show that at least some people who have been diagnosed with dyslexia do not show rapid adjustment of saccade amplitude in the context of the McLaughlin-type adaptation task used here. This failure to adapt motor output in response to a persistent visual error might result from either a failure to detect the error or an inability to appropriately use this information to alter saccade metrics. In this group, it remains unknown whether those that did not adapt also have cerebellar developmental differences that have been previously reported in some people with dyslexia [2, 44, 49, 69]. An important next step will be to investigate possible structure-function relationships by allying magnetic resonance imaging (MRI) measures of cerebellar integrity with psychophysical measures of adaptation in dyslexia. This association of cerebellar structural differences and adaptation deficits might serve to identify a specific sub-phenotype within the dyslexia population. Although given the large proportion of our participants that showed a lack of adaptation (90%), this may in fact be a fairly general issue in dyslexia. Note, however, in Table 1, that one of our participants with dyslexia did indeed show adaptation during the saccade adaptation paradigm. This participant was also the oldest participant in our DYS group (15.7 years old versus 15.2 for the next oldest). Although this study was not designed to assess the effects of age, considering that the oldest participant in the dyslexia group is the only one to show saccadic adaptation, one might be tempted to surmise that there is recovery of function with development. However, given that all the other dyslexia participants did not adapt, this interesting question simply cannot be addressed here; future investigation with larger samples spanning a greater age range will be needed to determine if there are developmental effects on saccadic adaptation in dyslexia.

Saccadic dysmetria that remains uncorrected through adaptation could lead to a spatial misalignment of auditory and visual stimuli which is critical for learning the pairing between orthographic tokens and phonemic utterances [4]. In future work, if saccade adaptation is to prove useful as a predictive measure of the risk of developing dyslexia, it will be important to map the development of this ability in considerably younger children than were assessed here. Early identification of children at risk for dyslexia could prove to be particularly useful since remediation of dysfluent reading can be difficult [70–72] particularly once children have advanced beyond elementary school [73]. To the extent that saccadic adaptation proves to be predictive of reading difficulties in this population, it provides a relatively quick and non-invasive tool for targeted corrective action.

## Conclusions

Saccadic adaptation is impaired in at least some portion of children diagnosed with developmental dyslexia compared to age-matched, typically developing children. This may result, in part, from the observed structural and functional differences in the cerebella of people with dyslexia [2, 44, 48, 49, 68, 69]. Saccadic adaptation has the potential to facilitate early diagnosis of dyslexia in order to initiate earlier remediation and provide better outcomes for this population.

## Abbreviations

ADHD: Attention deficit hyperactivity disorder
DYS: Dyslexia
TD: Typically developed
